# Risk Factors for Postoperative Cerebrospinal Fluid Fistulas After Craniotomy and Craniectomy: A Systematic Review and Meta-Analysis

**DOI:** 10.1007/s00701-025-06685-3

**Published:** 2025-10-03

**Authors:** Matteo Palermo, Fabio Zeoli, Valid Rastegar, Carmelo Lucio Sturiale, Francesco Signorelli

**Affiliations:** Department of Neurosurgery, Fondazione Policlinico Universitario A. Gemelli IRCCS, Università Cattolica del Sacro Cuore, Rome, Italy

**Keywords:** Cerebrospinal fluid leak, Craniotomy, Craniectomy, Postoperative complications, Dural closure

## Abstract

**Introduction:**

Postoperative CSF fistulas are a common complication in cranial neurosurgery. While efforts to prevent CSF leaks typically focus on surgical technique, it remains unclear whether tumor location, type of pathology, or specific closure methods independently influence fistula development. Clarifying these risk factors is essential to guide intraoperative decision-making and improve patient outcomes.

**Methods:**

We conducted a systematic search limited to peer-reviewed studies published in English on multiple databases. The search algorithm retrieved 1,348 results. After the exclusion phase, we included 26 comparative studies in the final analysis, collectively reporting data on 8,248 patients who underwent either craniotomy or craniectomy. After a systematic review, we performed a meta-analysis when sufficient data were available from multiple studies for a specific risk factor.

**Results:**

Infratentorial surgeries had a higher CSF leak rate (7.9%) than supratentorial ones (4.6%). Tumor surgeries showed greater risk than vascular procedures (OR: 1.82). Primary closure had a higher leak rate (12.3%) compared to patch grafts (8.5%). Watertight closure showed a trend toward fewer leaks than non-watertight closure, though not statistically significant. CSF leaks were strongly associated with postoperative infections (34.1%).

**Conclusion:**

Infratentorial location, tumor surgery, and sural closure increase the risk of postoperative CSF leaks. Patch grafts and watertight techniques lower this risk. Given the strong association with infections, preventing CSF leaks is essential to improve surgical outcomes.

## Introduction

Cerebrospinal fluid (CSF) leakage is a known complication of cranial neurosurgery that occurs when the dura mater does not seal completely after surgery. CSF can then escape through the dural defect, leading to a pseudomeningocele (fluid collection under the incision) or an external csf fistula (e.g. drainage of clear fluid from the wound, nose, or ear, depending on the surgical approach).

After craniotomies, CSF leaks incidence rates range from roughly 1% to 10% in most series reported in the literature [6, 16]. The risk of CSF leakage depends on the surgical context, locations and surgical techniques. For example, skull base procedures like transsphenoidal surgery historically reported 4% leak rates, whereas posterior fossa surgeries have shown incidence rates up to 20–30% [17].

Nowadays, the importance of a watertight dural closure is universally recognized and improved reconstruction methods have reduced these numbers. [2].

The postoperative CSF leak is more than just a nuisance as it can significantly worsen patient outcomes. The presence of CSF leakage is linked to severe infection risk as CSF outflow creates a pathway for bacteria, ultimately leading to superficial wound infection, meningitis, or brain abscess. This may lead to poor wound healing, post-operative hydrocephalus, prolonged hospitalization and need for reoperation.

Approximately 20–25% of patients with a postoperative leak may require an invasive intervention to address it, ranging from percutaneous taps and lumbar CSF diversion to reoperation. Overall, management of a leak often involves interventions that can be burdensome for the patient[11].

Despite the incidence and impact of this type of complication worldwide, the experiences reported in literature address the problem in an often disorganized, rarely reproducible and inhomogeneous manner. It is therefore difficult to objectively quantify the risk of CSF leaks based on patients and procedures features.

The aim of our study was to systematically review the literature and carry out a meta-analysis of possible risk factors of CSF leaks in patients undergoing craniotomy and craniectomy.

## Methods

**Fig. 1:**
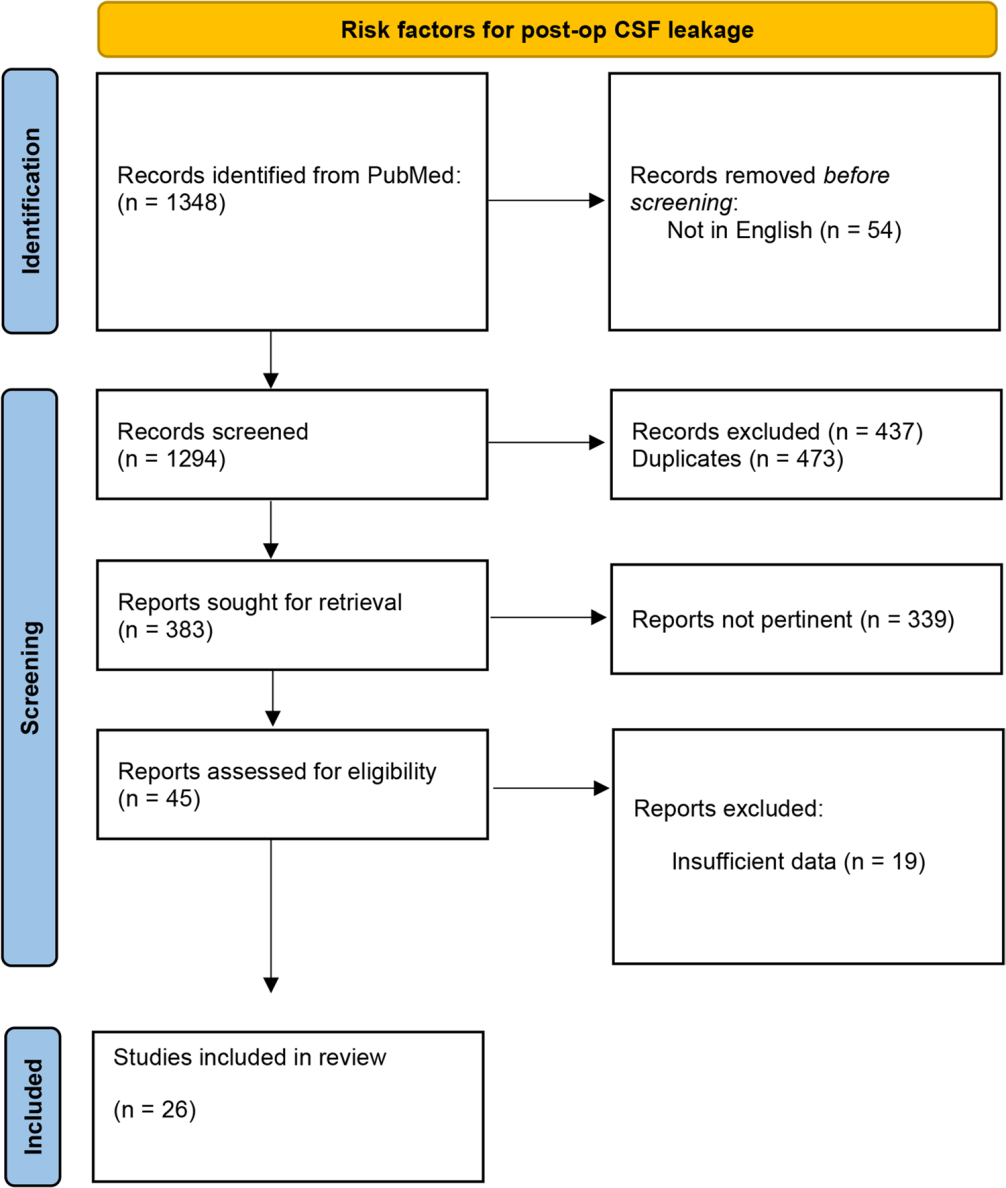
PRISMA 2020 flow diagram for new systematic reviews

This review was performed according to the PRISMA (Preferred Reporting Items for Systematic Reviews and Meta-Analyses) 2020 guidelines[27]. The PICO framework (Population: Craniotomy and Craniectomy patients; Intervention: CSF fistula; Comparison: Non-CSF fistula; Outcome: risk factors) was used to formulate the research question (Fig. 1).

### Search strategy

Two Authors (MP and VR) performed a comprehensive search on PubMed/MEDLINE and Scopus databases to identify relevant studies comparing people with and without CSF fistula using the search terms: ((" craniectomy" OR "craniotomy") AND ("CSF leak" OR "CSF leakage" OR "CSF fistula")). The search was updated to February 24th 2025, with no time limit. A forward search on references of the retrieved articles was also performed to increase the search power.

### Study Selection

The search was limited to peer-reviewed studies published in English with no time limit. Only papers containing quantitative data were included. Other inclusion criteria were: studies comparing patients with and without CSF fistula, cohort studies and case series with a minimum of 10 patients that reported outcomes in patients with and without CSF fistula. Eligible studies had to include a post-operative follow-up period, regardless of whether they were conducted prospectively or retrospectively. Randomized controlled trials (RCTs) were also included when available. Studies with insufficient patient numbers, lacking clear outcome reporting, or without follow-up data were excluded. Studies with insufficient patient numbers, lacking clear outcome reporting, or without follow-up data were excluded. Review papers, papers not reporting separate data for CSF and non-CSF fistula group, and papers discussing rinhorrea, otorrrhea, or pseudomeningocele were excluded, as the study’s specific focus was on postoperative CSF fistulas, typically referring to surgical wound leaks, rather than leaks through natural openings or fluid collection without a fistulous tract. Furthermore, studies that did not provide follow-up data were excluded, as they failed to meet the predefined inclusion criteria.

Three authors (MP and VR and FZ) independently screened titles and abstracts of the articles retrieved by the search algorithm and selected studies according to the inclusion or exclusion criteria. After the exclusion of ineligible articles, full texts of the remaining studies were assessed for eligibility according to the same criteria (Fig. 1). Disagreements were resolved with the senior authors (FS and CLS) through a new reading of the article and collegial re-evaluation of the extracted data.

### Data extraction

Initially, we collected data on the demographics of the patients and have reported them in Table 1. Then, we stratified data by surgical approach (supratentorial vs. infratentorial), and we further categorized procedures based on the type of intervention, specifically distinguishing tumor resections from vascular and other types of surgeries. This stratification was designed to investigate whether procedural characteristics commonly associated with dural-based tumors influence the risk of CSF leakage in other contexts (Table 2). Then, we calculated the prevalence rates for each intervention and reported them in Table 3. Finally, we stratified the data by tumor etiology and reported the results in Table 4.

**Table 1:** Summary of demographic data collected from included studies

Authors, Year	M	F	Comorbidities	Mean Age	
Guyolla, 2022	131	158	Diabetes (13); HTN (45)	N/A	
Afathy, 2024	204	336	N/A	N/A	
Khan, 2020	44	90	N/A	N/A	
Theys, 2018	3	7	N/A	51.3	
Wang, 2022	345	353	N/A	49.3	
Stieglitz, 2012	213	207	N/A	N/A	
Sathaporntheera, 2020	91	194	Hypertension (81);Dyslipedemia (72); Hypothyroidism (5); Smoke (25)	49.8	
Eichberg, 2018	N/A	N/A	N/A	57.2	
George, 2017	128	233	N/A	53.1	
Hutter, 2014	109	120	Diabetes (16); Allergy (48)	N/A	
Basu, 2016	N/A	N/A	N/A	N/A	
Jeswani, 2015	19	15	N/A	45.3	
Kim, 2013	51	106	N/A	50	
Kryzanski, 2007	N/A	N/A	N/A	N/A	
Kshettry, 2011	6	6	N/A	N/A	
Legnani, 2013	66	86	N/A	42.6	
Makarenko, 2015	12	23	N/A	57.6	
Marx, 2021	5	8	N/A	31.2	
Matano, 2025	15	19	N/A	60.1	
Kinaci, 2022	1117	1193	Diabetes (226); Liver disease (43); Renal disease(111); Thyroid(150); Bleeding disorder(129); alcohol use (828); Smoke (461)	52	
Ribeiro, 2021	110	161	N/A	55.9	
Prell, 2011	5	6	N/A	40	
Roberti, 2001	33	128	N/A	N/A	
Alwadei, 2019	107	109	N/A	44.55	
Lepski, 2021	N/A	N/A	N/A	52.7	
Sastry, 2022	98	171	Hypertension (64); Diabetes (19); Smoke (41)	39.7

**Table 2:** Summary of meta-analytical data collected from included studies

Authors. Year	Study design	N. patients		Overall N. of patients	Location of intervention (n. pts with CSF leak)		Type of surgey (n. pts with CSF leak)			Dura Closure technique (n. pts with CSF leak)		Watertight (n. pts with CSF leak)		Infection (n. pts with CSF leak)
		Craniotomy	Craniectomy		Infratentorial	Supratentorial	Tumor	Vascular	Trig Neur	Primary closure	Dural patch closure	Ye**s**	No	
**Guyolla, 2022**	**P**	**181**	**108**	**289**	**97(10)**	**184(24)**	**257 (34)**	**15 (0)**	**N/A**	**99 (8)**	**189 (28)**	**263 (29)**	**25 (7)**	**29 (19)**
**Afathy, 2024**	**R**	**540**	**N/A**	**540**	**N/A**	**N/A**	**469 (34)**	**N/A**	**71 (2)**	**N/A**	**N/A**	**N/A**	**N/A**	**N/A**
** Khan, 2020**	**R**	**134**	**N/A**	**134**	**N/A**	**N/A**	**N/A**	**N/A**	**N/A**	**N/A**	**134 (5)**	**N/A**	**N/A**	**N/A**
** Theys, 2018**	**R**	**10**	**N/A**	**10**	**10 (0)**	**N/A**	**1 (0)**	**9(0)**	**N/A**	**2(0)**	**8(0)**	**N/A**	**N/A**	**N/A**
** Wang, 2022**	**R**	**698**	**N/A**	**698**	**N/A**	**N/A**	**N/A**	**N/A**	**N/A**	**N/A**	**N/A**	**423 (41)**	**275 (31)**	**N/A**
**Stieglitz, 2012**	**R**	**420**	**N/A**	**420**	**N/A**	**N/A**	**420 (19)**	**N/A**	**N/A**	**N/A**	**420 (19)**	**N/A**	**N/A**	**N/A**
**Sathaporntheera, 2020**	**R**	**206**	**80**	**286**	**286 (40)**	**N/A**	**204 (29)**	**N/A**	**42 (5)**	**N/A**	**N/A**	**285(14)**	**1(0)**	**N/A**
** Eichberg, 2018**	**R**	**151**	**N/A**	**151**	**18 (0)**	**102 (0)**	**151 (0)**	**N/A**	**N/A**	**N/A**	**151(0)**	**N/A**	**N/A**	**N/A**
** George, 2017**	**RCT**	**778**	**N/A**	**778**	**N/A**	**317 (21)**	**N/A**	**N/A**	**N/A**	**364(72)**	**362(61)**	**N/A**	**N/A**	**N/A**
** Hutter, 2014**	**RCT**	**243**	**N/A**	**243**	**N/A**	**N/A**	**N/A**	**N/A**	**N/A**	**121 (20)**	**108 (11)**	**N/A**	**N/A**	**N/A**
** Basu, 2016**	**RCT**	**8**	**N/A**	**8**	**8(3)**	**N/A**	**7(2)**	**1(1)**	**N/A**	**N/A**	**N/A**	**N/A**	**N/A**	**N/A**
** Jeswani, 2015**	**R**	**34**	**N/A**	**34**	**N/A**	**34 (0)**	**34 (0)**	**N/A**	**N/A**	**N/A**	**N/A**	**N/A**	**N/A**	**N/A**
** Kim, 2013**	**R**	**157**	**N/A**	**157**	**N/A**	**157(11)**	**N/A**	**N/A**	**N/A**	**113(5)**	**44(6)**	**92(11)**	**65(0)**	**N/A**
** Kryzanski, 2007**	**R**	**29**	**N/A**	**29**	**N/A**	**29(0)**	**29(0)**	**N/A**	**N/A**	**N/A**	**N/A**	**N/A**	**N/A**	**1(0)**
** Kshettry, 2011**	**R**	**N/A**	**12**	**12**	**N/A**	**12(3)**	**12 (3)**	**N/A**	**N/A**	**N/A**	**N/A**	**N/A**	**N/A**	**12(3)**
** Legnani, 2013**	**R**	**100**	**52**	**152**	**152 (8)**	**N/A**	**152(8)**	**N/A**	**N/A**	**N/A**	**N/A**	**N/A**	**N/A**	**N/A**
** Makarenko, 2015**	**R**	**35**	**N/A**	**35**	**N/A**	**35(0)**	**35(0)**	**N/A**	**N/A**	**N/A**	**N/A**	**N/A**	**N/A**	**N/A**
** Marx, 2021**	**R**	**13**	**N/A**	**13**	**N/A**	**13(2)**	**13(2)**	**N/A**	**N/A**	**N/A**	**N/A**	**N/A**	**N/A**	**2(0)**
** Matano, 2025**	**R**	**34**	**N/A**	**34**	**N/A**	**N/A**	**N/A**	**34(0)**	**N/A**	**N/A**	**N/A**	**N/A**	**N/A**	**N/A**
** Kinaci, 2022**	**R**	**2310**	**N/A**	**2310**	**540(63)**	**N/A**	**1297(102)**	**672(30)**	**N/A**	**N/A**	**1016(95)**	**N/A**	**N/A**	**N/A**
**Ribeiro, 2021**	**R**	**271**	**N/A**	**271**	**N/A**	**N/A**	**271(12)**	**N/A**	**N/A**	**N/A**	**N/A**	**N/A**	**N/A**	**N/A**
** Prell, 2011**	**R**	**11**	**N/A**	**11**	**11(0)**	**N/A**	**10(0)**	**1(0)**	**N/A**	**N/A**	**N/A**	**N/A**	**N/A**	**N/A**
**Roberti, 2001**	**R**	**161**	**N/A**	**161**	**N/A**	**N/A**	**161 (22)**	**N/A**	**N/A**	**N/A**	**N/A**	**N/A**	**N/A**	**N/A**
** Alwadei, 2019**	**R**	**216**	**N/A**	**216**	**N/A**	**216 (8)**	**N/A**	**N/A**	**N/A**	**N/A**	**N/A**	**114 (2)**	**102 (6)**	**N/A**
** Lepski, 2021**	**R**	**987**	**N/A**	**987**	**N/A**	**N/A**	**N/A**	**N/A**	**N/A**	**N/A**	**N/A**	**N/A**	**N/A**	**53(13)**
** Sastry, 2022**	**R**	**185**	**84**	**269**	**269(10)**	**N/A**	**82(7)**	**28(0)**	**N/A**	**N/A**	**N/A**	**N/A**	**N/A**	**N/A**

**Table 3:** Summary of prevalence

		**Pts**	**CSF pts**	**Prevalence (% CSF leak)**	**95% CI (%)**	**No of studies**	**p-value**	**I**^**2**^** (%)**
	**Included studies**					26		
	**Total no of patients**	8248				26		
	**Craniotomy**	7912				26		
	**Craniectiomy**	336				5		
**LOCATION**	**Infratentorial**	1391	134	7.90	4.30—11.60	9	< 0.001	78.72
	**Supratentorial**	1099	69	4.6	2—7.3	10	< 0.001	80.7
**TYPE OF SURGERY**	**Tumor**	3605	274	6.6	4.1—9.0	18	< 0.001	89.33
	**Vascular**	760	31	3.30	0.70 −5.90%	7	0.188	31.46
	**Trigeminal Neuralgia**	113	7	6.20	−2.40–14.80	2	0.091	65.1
**TYPE OF CLOSURE**	**Primary Closure**	699	105	12.30	4.60—19.90	5	< 0.001	87.86
	**Dural Patch closure**	2432	225	8.5	4.4—12.7	9	< 0.001	95.09
**CLOSURE TECHNIQUE**	**Watertight**	1177	97	7.50	3.60—11.30	5	< 0.001	86.25
	**No Watertight**	468	44	8.5	1.5- 15.5	5	< 0.001	87.51
**POST-OP OUTCOME**	**Infection**	97	35	34.10	12.2—56.0	5	0.002	75.84

**Table 4:** Summary of incidence stratified by tumor type

Author, Year	Meningioma	Glioma	Metastasis	
**Guyolla, 2022**	N/A	N/A	N/A	
**Afathy, 2024**	157 (8)	N/A	49 (4)	
** Khan, 2020**	N/A	N/A	N/A	
** Theys, 2018**	1(0)	N/A	N/A	
** Wang, 2022**	N/A	N/A	N/A	
**Stieglitz, 2012**	N/A	N/A	N/A	
**Sathaporntheera, 2020**	51 (4)	N/A	N/A	
** Eichberg, 2018**	31(0)	N/A	34(0)	
** George, 2017**	N/A	N/A	N/A	
** Hutter, 2014**	N/A	N/A	N/A	
** Basu, 2016**	N/A	N/A	N/A	
** Jeswani, 2015**	N/A	N/A	N/A	
** Kim, 2013**	N/A	N/A	N/A	
** Kryzanski, 2007**	29(0)	N/A	N/A	
** Kshettry, 2011**	5 (1)	3(1)	1(0)	
** Legnani, 2013**	N/A	N/A	N/A	
** Makarenko, 2015**	35(0)	N/A	N/A	
** Marx, 2021**	N/A	N/A	N/A	
** Matano, 2025**	N/A	N/A	N/A	
** Kinaci, 2022**	N/A	N/A	N/A	
**Ribeiro, 2021**	N/A	N/A	N/A	
** Prell, 2011**	1(0)	7(1)	2(0)	
**Roberti, 2001**	161 (22)	N/A	N/A	
** Alwadei, 2019**	N/A	N/A	N/A	
** Lepski, 2021**	N/A	N/A	N/A	
** Sastry, 2022**	N/A	N/A	N/A	
TOTALS	470(34)	10(2)	94(4)	

### Presentation of data and statistical analysis

After a systematic review, we performed a meta-analysis when sufficient data were available from multiple studies for a specific risk factor. This allowed for odds ratios (OR) calculation comparing CSF leaks for a limited number of risk factors (dural technique and watertightness). Statistical analyses were performed using OpenMetaAnalyst software (http://www.cebm.brown.edu/openmeta/), based on R and funded by the Agency for Healthcare Research and Quality (Rockville, MD, USA).

### Quality assessment (risk of bias)

**Fig. 2:**
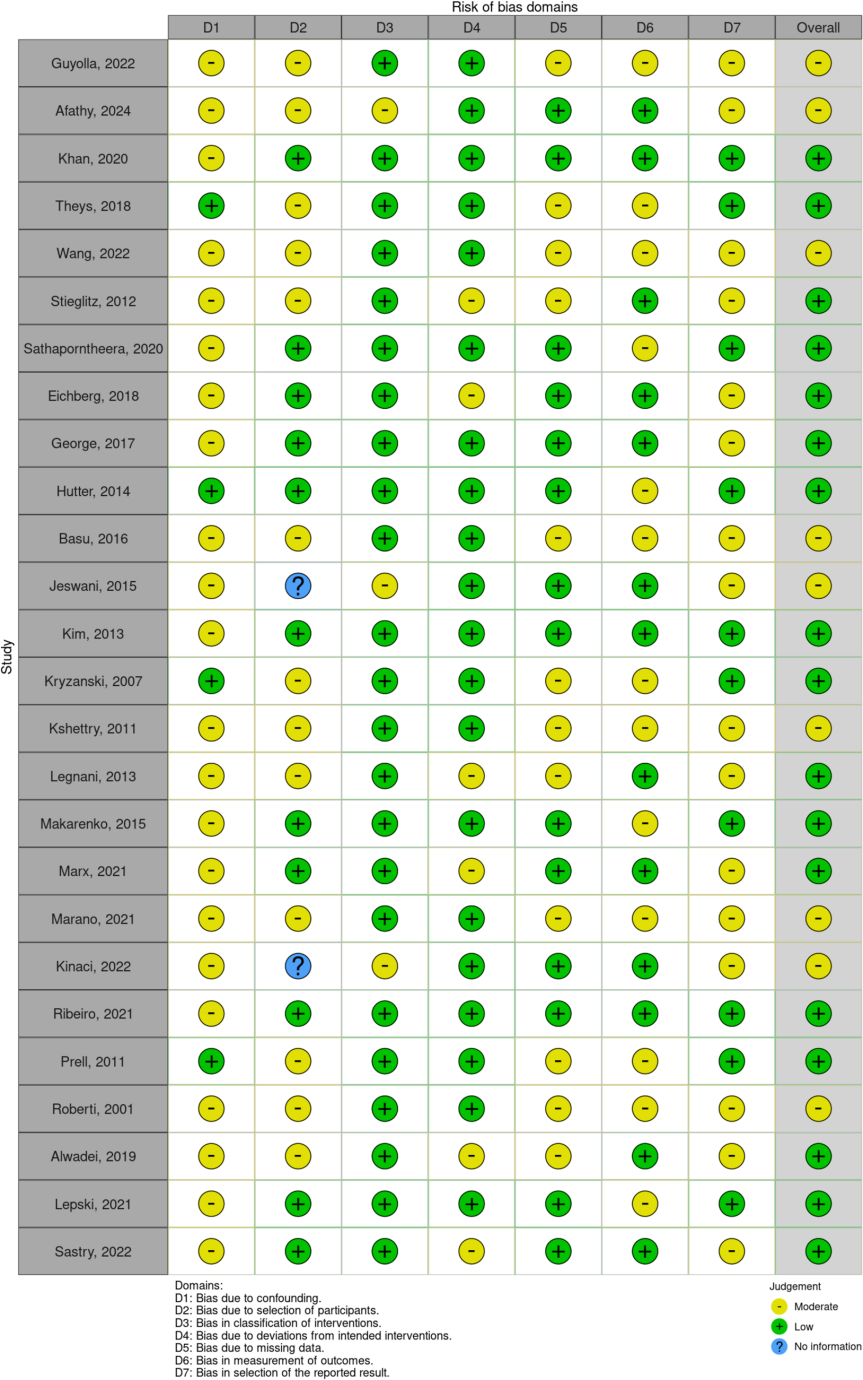
ROBINS-I V2 (Risk of Bias In Non-randomized Studies – of Interventions, Vers. 2)

The ROBINS-I V2 (Risk of Bias In Non-randomized Studies – of Interventions, Version 2) assessment tool along with the robins application (https://mcguinlu.shinyapps.io/robvis/) were used to evaluate study quality through visual representations (Fig. 2).

**Fig. 3:**
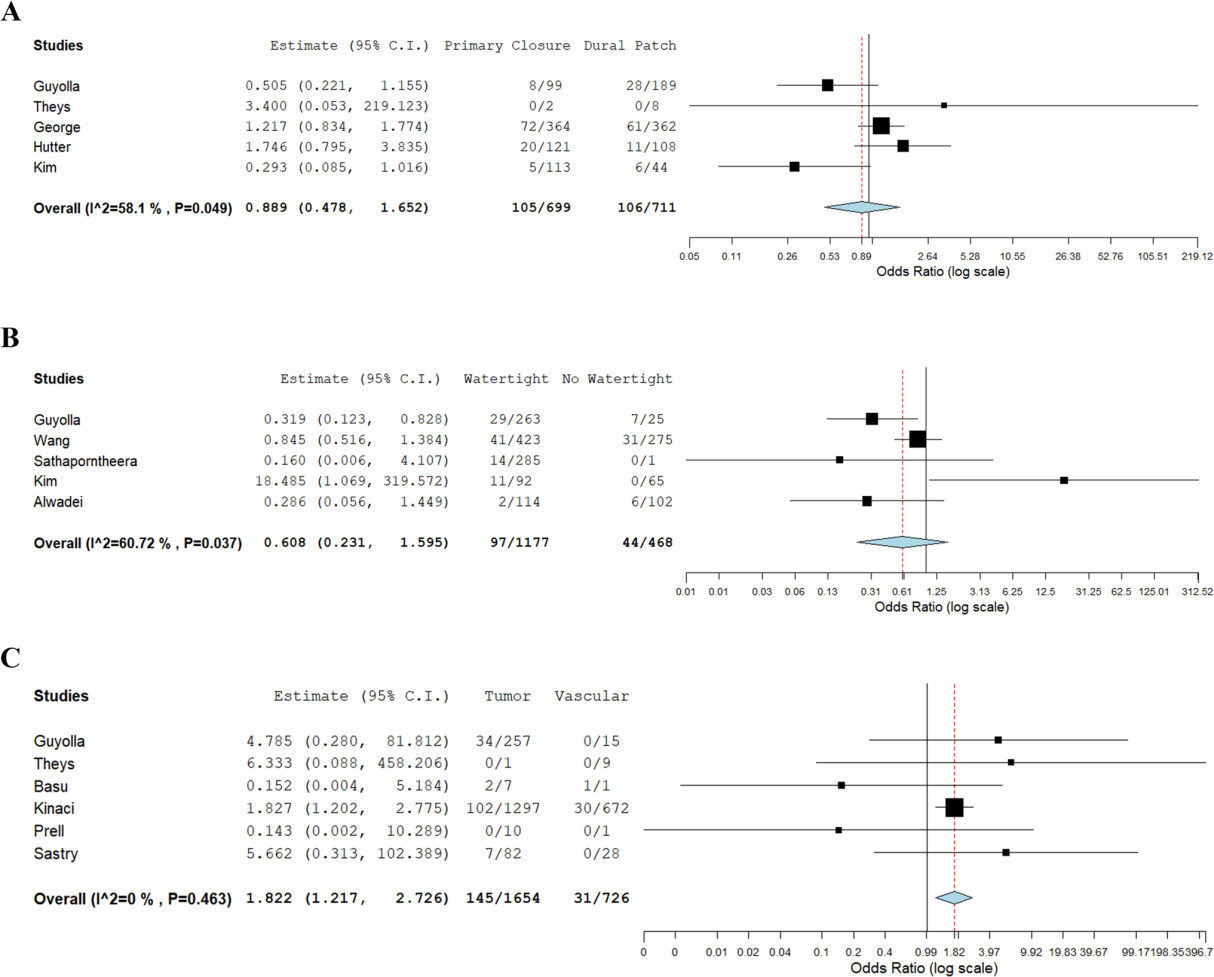
**A** forest plot of odds ratio Primary Closure Vs Dural Patch, **B** forest plot of odds ratio Watertight Vs Non-Watertight, **C** forest plot of odds ratio Tumor Vs Vascular

## RESULTS

The search algorithm retrieved 1,348 results. The initial screening process excluded irrelevant articles based on predefined inclusion and exclusion criteria. Specifically, 54 documents were excluded because not in English, 473 were duplicates, 437 were unrelated to the research question, 178 examined only CSF fistula patients without comparative data, 73 centered on revision surgeries, 83 discussed only surgical techniques and 5 were letters to the editor.

The secondary screening stage aimed to isolate studies that stratified outcomes based on the presence or absence of postoperative CSF fistula. During this stage, articles with insufficient data and reviews were also excluded (n = 19).

Overall, we included 26 comparative studies in the final analysis (Fig. 1; Table 1), whose quality was assessed using ROBINS-I V2 tool for risk of bias evaluation (Fig. 2).

The study selection process was documented using the PRISMA 2020 flowchart, outlining the stages of identification, screening, eligibility assessment, and final inclusion (Fig. 1).

### Systematic review (qualitative data)

A total of 26 studies, collectively reporting data on 8,248 patients who underwent either craniotomy or craniectomy are included in this systematic review. Overall, 7,912 craniotomies and 336 craniectomies were analyzed across all studies.

In total, from the studies that provided gender data, 2,945 males and 3,254 females were included. Age data were reported in 18 out of 27 studies, all as mean ages. The mean age of participants across studies generally ranged from early 30 s to early 60 s. The lowest mean age was 31.2 years[24], and the highest was 60.1 years [25], with a pooled average age centered around the late 40 s to early 50 s. Comorbidities were reported inconsistently across the studies. Common comorbidities included diabetes, hypertension, smoking, and alcohol use. Full details are provided in Table 1 [1, 5, 14, 19, 22, 29, 30].

Postoperative cerebrospinal fluid fistula was reported in both supratentorial and infratentorial procedures, with infratentorial surgeries showing 134 cases of CSF leakage out of 1,391 procedures (7.9%, 95% CI: 4.30%—11.60%, P < 0.001, I^2^ = 78.72)[5, 7, 10, 16, 21, 28, 32, 33]. In supratentorial procedures, 69 CSF leaks were documented out of 1,099 surgeries (4.6%, 95% CI: 1.2%—7.3%. P < 0.001, I^2^ = 80.7) [3, 7, 9, 10, 13, 15, 19, 20, 23, 24, 34].

When stratified by the primary surgical indication, tumor surgeries accounted for the largest number of cases with 3,586 procedures, within which 254 postoperative CSF leaks were reported, giving an incidence rate of 6.60% (95% CI: 4.1%—9.0%, P < 0.001, I^2^ = 89.33) [1, 5, 7, 10, 13, 16, 19–21, 23, 24, 26, 28–33] Vascular surgeries were represented by 760 cases, with 31 CSF leaks occurring (3.30%,95% CI: 0.70% −5.90%,P < 0.001, I^2^ = 31.46). Among 113 trigeminal neuralgia cases, 7 developed postoperative CSF leaks (6.20%,95% CI: −2.40%−14.80%, P < 0.001, I^2^ = 65.1) [1, 32].

Additionally, closure technique was reported only in a subset of studies. Primary dural closure alone was documented in 699 cases, with a CSF-leak prevalence of 12.30% (95% CI: 4.60%—19.90%,P < 0.001, I^2^ = 87.86) [9, 10, 12, 15, 33]. Differently, among cases where a dural patch was used for closure, only 225 out of 2,432 surgeries developed postoperative CSF leaks, corresponding to a leak rate of 8.5% (95% CI: 4.4%—12.7%, P < 0.005, I^2^ = 95.09) [7, 9, 10, 12, 14–16, 29, 33].

Similarly, among 1,177 cases of watertight closure, 97 developed postoperative CSF leaks with a leak rate of 7.5% (95% CI: 3.60%—11.30%, P < 0.001, I^2^ = 86.25), while non-watertight closure resulted in 44 leaks out of a total of 468 documented cases, giving a leak rate of 8.40% (95% CI: 1.50%—15.30%, P < 0.001, I^2^ = 87.51) [3, 10, 15, 32, 34].

Comparable infection rates were reported only in 97 patients. Of those, 35 developed a postoperative CSF fistula, yielding a postoperative infection-associated leak rate of 34.1% (95% CI: 12.2%—56.0%,P = 0.002, I^2^ = 75.84) [10, 19, 20, 22, 24].

### Meta-analysis (quantitative data)

A total of five studies compared watertight versus non-watertight dural closure. Across these studies, 97 CSF leaks occurred in 1,177 patients with watertight closure, and 44 CSF leaks in 468 patients without watertight closure. The pooled odds ratio was 0.608 (95% CI: 0.231–1.595), with heterogeneity observed among studies. (P < 0.001; I^2^ = 60.72%) (Fig. 3A).

The studies with available data for a metanalysis comparing primary closure to dural closure techniques, demonstrated a pooled odds ratio of 0.889 (95% CI: 0.478–1.652), with moderate heterogeneity (P < 0.001; I^2^ = 58.1%) (Fig. 3B).

Furthermore, studies with available data were meta-analyzed to compare the odds of developing a CSF leak in tumor versus vascular neurosurgery. The pooled odds ratio was 1.822 (95% CI: 1.217–2.726), with no observed heterogeneity (I^2^ = 0%, P < 0.001) (Fig. 3C).

## DISCUSSION

CSF fistulas remain a significant postoperative complication in cranial neurosurgery, often resulting in prolonged hospitalization, increased infection risk, and need for additional interventions. Our findings provide a comprehensive overview of CSF fistula risk across different surgical contexts to help optimize preventive strategies and improve patient outcomes.

### Infratentorial vs Supratentorial CSF leak rates

Our meta-analysis identified clear patterns in postoperative CSF fistula risk across different surgical contexts. As expected, infratentorial procedures showed a higher incidence of CSF leaks (7.90%) compared to supratentorial operations (4.60%). This finding suggests that tumor location might influence fistula development. The confined anatomy of the posterior fossa and proximity to major CSF cisterns likely predispose infratentorial surgeries to dural closure challenges and occult leaks. Our observations are consistent and add quantitative detail to prior reports in the neurosurgical literature. The higher propensity of infratentorial surgeries to develop CSF leaks is well documented. Historically, posterior fossa craniotomies have been reported to have CSF leak rates up to 20–30%, much higher than rates in other cranial locations. For example, Cheong et al. found that infratentorial craniotomies were an independent predictor of post-craniotomy CSF leakage, whereas supratentorial cases had significantly fewer leaks. A recent large multi-center study by Kinaci et al. (2023)[16] likewise confirmed that infratentorial surgery roughly doubles the risk of an incisional CSF leak. In their cohort of 2,310 patients, 11.7% of infratentorial craniotomies resulted in a leak versus only 5.8% of supratentorial procedures. Our analysis of prevalence (7.90% vs 4.6%) aligns closely with these figures, reflecting improvements over historical rates yet preserving the relative difference. The anatomical and technical challenges of posterior fossa surgery, such as the need to open the cisterna magna, the dependent gravitational position of the wound, and often the use of a craniectomy rather than replacement, likely explain the increased leak tendency. Another important point is that comparisons between primary closure and dural substitutes may be biased: patches are often applied when a visible leak or dural defect is present, meaning these cases are inherently higher risk, while suture-only cases may appear safer simply because no leak was evident at closure. In addition, the structural characteristics of the native dura mater vary significantly depending on the anatomical location, and these differences may influence the risk of CSF fistula formation. The supratentorial dura is generally thicker, more fibrous, and easier to suture securely, which may facilitate a more reliable primary closure. In contrast, the infratentorial dura, particularly in the posterior fossa, is thinner, more delicate, and often subjected to higher CSF pressure due to its proximity to the basal cisterns and fourth ventricle, which are open while approaching the posterior fossa tumors. This makes watertight closure more challenging in infratentorial procedures, contributing to the higher observed leak rates in posterior fossa surgeries.

### Type of surgery and CSF leak rates

In terms of surgical indication, our finding that cerebrovascular cases have the lowest leak incidence is supported by prior data. Kinaci et al.[16] observed that vascular surgeries (e.g. for aneurysm or AVM) had the lowest baseline leak risk in their series. We found a similarly low rate (3.30%). Additionally, our metanalysis comparing CSF leak rates between tumor and vascular neurosurgical procedures yielded a pooled odds ratio of 1.822 (95% CI: 1.217–2.726), indicating that patients undergoing tumor surgeries have higher odds of developing CSF leaks compared to those undergoing vascular procedures. The analysis demonstrated no observed heterogeneity among the included studies (I^2^ = 0%, P < 0.001), suggesting consistent findings across the studies analyzed (Fig. 3C).

However, none of the studies reporting CSF leak rates after vascular and tumor surgeries explicitly addressed the underlying causes. We hypothesize that the lower leak rates observed in vascular procedures may be due to the typically small dural openings, which are often straightforward to close. However, it should also be noted that vascular approaches frequently involve opening of the basal cisterns, potentially exposing the surgical field and closure site to continuous CSF pulsatility. In contrast, procedures involving extensive posterior fossa exposure or significant bone removal are consistently associated with higher leak rates in the literature. In support to this idea, a recent analysis of 225 retrosigmoid craniotomies for cerebellopontine angle (CPA) tumors reported a 31% incidence of postoperative CSF leakage, underlining how challenging dural repair can be in skull base tumor cases[8]. The prevalence of CSF leak rate for tumor surgeries in our cohort was 6.6%, which is lower than the rate reported by Esposito et al., but higher than what has been described in the literature for gliomas and metastases[8]. This discrepancy may be explained by the broad range of tumor types and anatomical locations included in our analysis, many of which were supratentorial or convexity tumors that typically allow for more straightforward dural closure. Notably, the majority of tumor cases were meningiomas (470 cases, Table 3), which are well known to be associated with higher CSF leak rates due to extensive dural involvement. Excluding glioma cases (n = 10) due to their limited number, meningiomas demonstrated a CSF leak rate of 7.23% (34/470), whereas surgeries for metastases had a lower leak rate of 4.26% (4/94).

Based on the statistical calculations, our observed 6.2% CSF leak rate for microvascular decompression (MVD) in trigeminal neuralgia cases appears to fall within the expected range of moderate risk. While some series report lower rates (1–2%) when meticulous dural closure and bone reconstruction are performed, our rate is consistent with the notion that MVDs carry a leak risk higher than simple supratentorial procedures but lower than more extensive posterior fossa tumor resections. Further research with more consistent methodology may be needed to draw definitive conclusions.

### Primary closure vs Dural Patch

Perhaps the most striking result of our analysis was the nearly 1.5-fold difference in leak incidence between primary suture closure versus patch graft duraplasty (12.3% vs 8.5%). This suggests that a properly performed dural patch can almost entirely prevent postoperative CSF leakage. For instance, Cheong et al. compared primary suturing alone to suturing plus a dural only patch (Duraform) in 363 craniotomies. They found the CSF leak rate was reduced from 12.6% with suture-only to 5.1% with a patch graft. Similarly, they observed that adding a dural sealant significantly lowered the incidence of surgical site infection, in tandem with leak reduction. However, it is worth noting a subtle point: in the Kinaci et al.[16] series, the use of a dural substitute was paradoxically associated with higher leak risk on multivariate analysis. This paradox is likely reflected by the findings of our meta-analysis **(**Fig. 3A**)** on the odds of primary suture closure versus dural patching. In particular, the comparison (OR = 0.889, CI: 0.478–1.652, P < 0.001) showed statistically significant difference in CSF leak rates despite a moderate heterogeneity (I^2^ = 58.1%). This suggests that dural patching may offer a comparable safety profile to primary closure.

However, modern adjuncts like hydrogel sealants have been shown to improve the seal. For example, Auricchio et al.[4] noted that in CPA tumor surgeries, combining a heterologous dural patch with a hydroset bone repair significantly lowered leak rate compared to patch alone. Taken together, our findings disagree with the majority of the literature which instead supports that meticulous dural reconstruction is the most effective strategy for lowering the risk of CSF fistulas.

### Watertight vs Non-Watertight

Traditional neurosurgical teaching has long emphasized achieving a “watertight” closure. The analysis comparing watertight vs non-watertight dural closure (OR = 0.608. 95% CI: 0.231–1.595, P < 0.001) suggests a potential reduction in postoperative CSF leaks with watertight closures. While the p-value falls below the conventional threshold for significance, indicating a potential benefit, the wide confidence interval crossing 1.0 reflects uncertainty in the estimate and limits the strength of the conclusion. This discrepancy highlights that although watertight closure may be clinically favorable, the current evidence does not conclusively demonstrate a statistically significant advantage, and further high-quality studies are warranted to clarify its true impact.

### Infections in CSF-leak patients

Finally, even the strong association between CSF fistulas and postoperative infection in our analysis is well corroborated by prior studies. In our cohort, over one-third of patients with with an infection had a leak (34.1%; 95% CI: 0.122–0.056; P < 0.001, I^2^ = 75.84). In comparison, typical cranial surgery infection rates are much lower, usually in the range of 1–5% for clean elective craniotomies. This suggests that the presence of a CSF leak may increase infection risk by an order of magnitude. Indeed, Kinaci et al.[16] reported that patients with an incisional CSF leak had 15 times higher odds of developing a wound infection or meningitis than those without a leak. Perhaps the most compelling evidence comes from a prospective study by Kourbeti et al.[18]: they found that a postoperative CSF leak was an independent predictor of post-craniotomy meningitis with an OR of 48 (p < 0.001). In that study, every patient who developed meningitis had a clear route of CSF egress (either an incisional leak or an external CSF drain), emphasizing how critical a sealed dura is for infection prevention. Additionally, persistent CSF leakage may lead to dead space and fluid collections that foster bacterial growth. The literature also suggests a bidirectional relationship: not only do leaks promote infection, but infections such as meningitis can exacerbate leakage by impeding wound healing and increasing inflammation at the dural interface. Overall, our results and prior studies collectively underscore that a CSF fistula is not a benign complication; it is strongly linked to infectious morbidity and must be addressed promptly when identified.

### Limitations

While our meta-analysis provides a comprehensive overview of risk factors for post-craniotomy CSF fistulas, several limitations must be acknowledged. First, the analysis is based on pooled data from 26 comparative studies, which introduces heterogeneity in definitions and reporting. There was variability in how each study defined a “CSF leak”, some included only external incisional leaks, others may have included subgaleal pseudomeningoceles if they required intervention without clearly stating it. We excluded studies focusing on rhinorrhea/otorrhea and pseudomeningocele, as they represent distinct clinical manifestations, involving skull base defects with extracranial drainage or subcutaneous CSF collection without external leakage. Including them could have introduced heterogeneity and inflated or misrepresented overall CSF leak incidence in the context of postoperative cranial surgery.

A significant limitation of this analysis lies in the incomplete and inconsistent reporting of comorbidities across the included studies. Only 2 out of 26 studies provided sufficient detail to calculate the incidence of CSF leak in patients with specific comorbid conditions. In these studies, CSF leaks occurred in 17 out of 239 diabetic patients (7.1%) and 7 out of 45 hypertensive patients (15.6%). Additional reported rates included 3 out of 43 patients with liver disease (7.0%), 12 out of 111 with renal disease (10.8%), 9 out of 150 with thyroid disorders (6.0%), 9 out of 129 with bleeding disorders (7.0%), and 53 out of 828 individuals with alcohol use (6.4%). The remaining studies either reported comorbidities without linking them to clinical outcomes or omitted such data entirely. This lack of standardized and comprehensive reporting limits the ability to assess the true impact of individual comorbidities on CSF leak incidence. Furthermore, we tried to subclassified by tumor type (meningiomas, gliomas, metastases); corresponding CSF leak rates are reported in Table 3. However, given the markedly unbalanced subgroup sizes, comparative interpretation remains limited.

We focused on location, indication, and closure technique, but other contributors to CSF leak risk were not uniformly reported across studies, for instance, patient factors like body mass index, smoking status, or use of postoperative drains. Kinaci et al. suggests young age, obesity, and smoking all increased leak rates, but we could not assess these in our meta-analysis due to lack of sufficient data [16]. Future studies should conduct an age-stratification analysis for the onset of CSF-leaks. Additionally, none of the included studies analyzed the opening of the Similarly, operative duration and surgeon technique are hard to quantify but likely play a role, longer, more invasive surgeries presumably carry higher leak risk. Our analysis by broad categories (e.g., “tumor surgery” encompassing everything from small convexity meningiomas to large skull base schwannomas) may mask important nuances. The included papers were mostly retrospective observational studies with their own biases. Many were single-center experiences with relatively small numbers of leak events. Given the heterogeneity of the included studies and the large datasets analyzed, detailed information on the specific methods of dural closure could not be consistently collected. For this reason, direct head-to-head comparisons to determine the best technique are not feasible. Finally, as with any meta-analysis, statistical heterogeneity must be considered. Where we pooled data, we observed some inconsistency. This reflects genuine differences in practice patterns and patient populations. Such differences complicate direct comparisons. Despite these limitations, the consistency of our core findings with prior knowledge lends credibility to the conclusions. Future prospective studies or randomized trials would be valuable to more definitively quantify risk reductions. Until then, our compiled results provide the best available evidence to guide clinicians in identifying high-risk scenarios to prevent CSF fistulas and their complications.

## CONCLUSION

Location and surgical procedure both influence the risk of postoperative CSF fistula, with infratentorial procedures showing higher risks. Closure techniques play a crucial role, with path grafts reporting lower incidence of CSF leaks compared to primary closures. Additionally, CSF leaks are associated with a significant risk of infections. These findings underscore the importance to tailor the possible risks in advance to minimize complications and improve surgical outcomes, when facing with patients who need to undergo craniotomy or craniectomy.

## Data Availability

No datasets were generated or analysed during the current study.

## Abbreviations

CSF: Cerebrospinal Fluid
OR: Odds Ratio
MVD: Microvascular Decompression
CPA: Cerebellopontine Angle
RCT: Randomized Controlled Trial
CI: Confidence Interval
